# Metastatic Epithelioid Pleomorphic Liposarcoma in the Brain: A Case Report

**DOI:** 10.7759/cureus.50066

**Published:** 2023-12-06

**Authors:** Tengfei Wang, Kiersten L Waworuntu, Frank Y Shan

**Affiliations:** 1 Pathology, Baylor Scott & White Health, Temple, USA; 2 Pathology and Neurosurgery, Baylor Scott & White Health, Temple, USA

**Keywords:** histomorphology, mdm2, brain metastasis, epithelioid variant, pleomorphic liposarcoma

## Abstract

Metastatic soft tissue sarcoma (STS) to the brain is rare, and metastatic pleomorphic liposarcoma (PLPS) to the brain is even rarer. We present the case of a 76-year-old male with an incidental finding of a brain mass on a brain magnetic resonance imaging (MRI) following a head injury. Additionally, multiple pulmonary nodules and a right gluteal mass were discovered. A core biopsy of the right gluteal mass revealed an epithelioid malignant neoplasm expressing transcription factor enhancer 3 (TFE3) by immunohistochemistry (IHC). Subsequently, the left middle fossa brain mass was removed and found to be an epithelioid PLPS, which was positive for TFE3 by IHC but lacked TFE3 rearrangement by fluorescence in situ hybridization (FISH), and negative for murine double minute (MDM2) amplification by FISH. The diagnosis of epithelioid PLPS mainly relies on histomorphology. This paper discusses the clinicopathological correlation of PLPS, including the epithelioid variant, with a focus on cases with brain metastases.

## Introduction

Soft tissue sarcomas (STSs) constitute 75% of all sarcomas, comprising less than 1% of malignancies in adults. Importantly, mortality rates for STSs have been rising in most countries for both genders [1]. Among STSs, approximately 15-20% are liposarcomas (LPS) [2,3].

The latest WHO classification categorizes LPS into five subtypes: well-differentiated LPS (WDLPS), de-differentiated LPS (DDLPS), myxoid LPS (MLPS), pleomorphic LPS (PLPS), and myxoid pleomorphic LPS (MPLPS) [4]. PLPS is the rarest, accounting for less than 5% of all liposarcomas [5]. The majority of PLPS cases are located in deep tissues, grossly firm and nodular with a white to yellow cut surface, and often exhibit hemorrhagic and necrotic areas, and microcystic degeneration. Microscopically, PLPS displays numerous pleomorphic lipoblasts mixed with pleomorphic spindle and/or epithelioid cells. PLPS can stain positively for non-specific markers such as smooth muscle actin (SMA), desmin, and cluster of differentiation (CD)34, adipophilin, b cell lymphoma-2 (BCL-2), survivin, p16, matrix metalloproteinase 2 (MMP2), and MMP9 [6,7]. PLPS is negative for murine double minute (MDM2) and cyclin-dependent kinase 4 (CDK4), which are markers of WDLPS and DDLPS [7]. The genomic profile is non-specific, including the loss of the tumor protein p53 (p53) and retinoblastoma (Rb). The differential diagnoses for PLPS encompass DDLPS with homologous lipoblastic differentiation, undifferentiated pleomorphic sarcoma, and pleomorphic non-mesenchymal tumors [7]. A subtype of PLPS, the epithelioid variant, accounts for 10% of pure epithelioid cases and up to 25% of cases with epithelioid features [5].

Here, we reported a case of gluteal epithelioid PLPS with brain metastasis (BM) and positive expression of transcription factor enhancer 3 (TFE3) by immunohistochemistry (IHC), but negative for MDM2 amplification by fluorescence in situ hybridization (FISH). While several non-specific markers showed positivity in this case, the primary diagnosis was predominantly based on histomorphology.

## Case presentation

A 76-year-old male presented for worsening headaches, diplopia, blurry vision, and left ptosis following a head injury. A brain/orbit magnetic resonance imaging (MRI) showed a 3.2 cm left middle fossa brain mass with vasogenic edema that extended to his left optic nerve. Thoracic and abdominal computed tomography (CT) scans showed multiple bilateral pulmonary nodules (up to 1.5 cm) consistent with metastatic lesions. A physical examination found a 7.0 cm right gluteal soft tissue mass that existed for approximately a month and was painful only when he sat.

**Figure 1:**
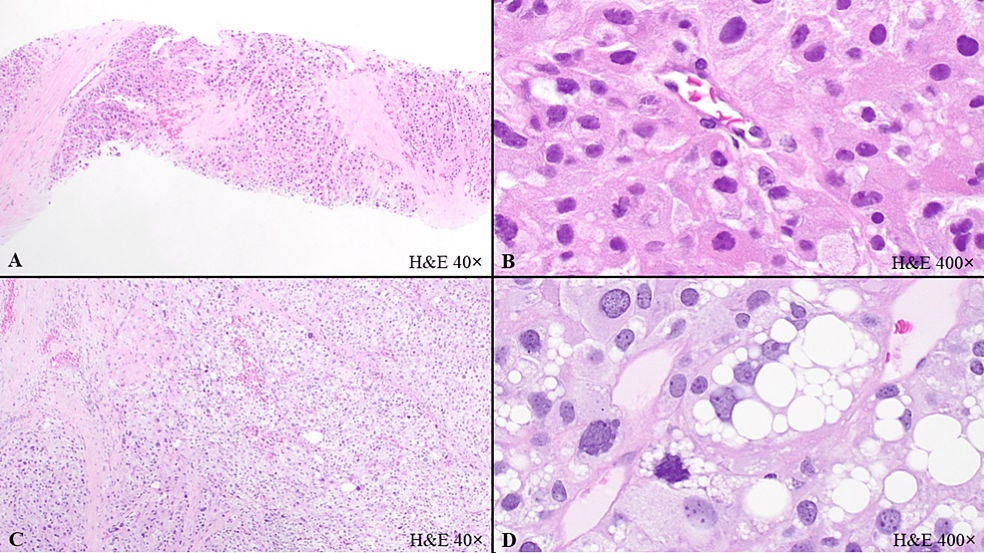
Histological features of epithelioid pleomorphic liposarcoma in the gluteus and brain. (A) and (B) Gluteal mass core biopsy with epithelioid pleomorphic liposarcoma. Tumor nests with a collagenous matrix (A, H&E, 40x), and pure epithelioid cytomorphology (B, H&E, 400x). (C) and (D) Brain mass resection with metastatic epithelioid pleomorphic liposarcoma. Tumor nests and sheets with collagenous matrix (C, H&E, 40x), and numerous pleomorphic lipoblasts (D, H&E, 400x).

A CT-guided core biopsy of the gluteal mass was performed. The core biopsy showed a malignant neoplasm composed of epithelioid cells with moderate nuclear pleomorphism and granular cytoplasm arranged in solid nests and sheets separated by a collagenous matrix, with multiple foci of necrosis (Figures 1A, 1B). No obvious lipoblastic differentiation was seen. By IHC the tumor cells were diffusely positive for TFE3, focally positive for epithelial membrane antigen (EMA), and negative for pankeratin (AE1/AE3, OSCAR), S100, human melanoma black 45 (HMB45), Melanoma Antigen Recognized by T cells 1 (MART1), SRY-related HMG-box 10 (SOX10), paired box gene 8 (PAX8), CD31, CD34, smooth muscle actin (SMA), desmin, synaptophysin, arginase-1, calretinin, inhibin, and CD68. Periodic acid-Schiff (PAS) stain with and without diastase highlighted intracytoplasmic glycogen. The diagnosis of malignant epithelioid neoplasm with TFE3 expression by IHC was rendered.

**Figure 2:**
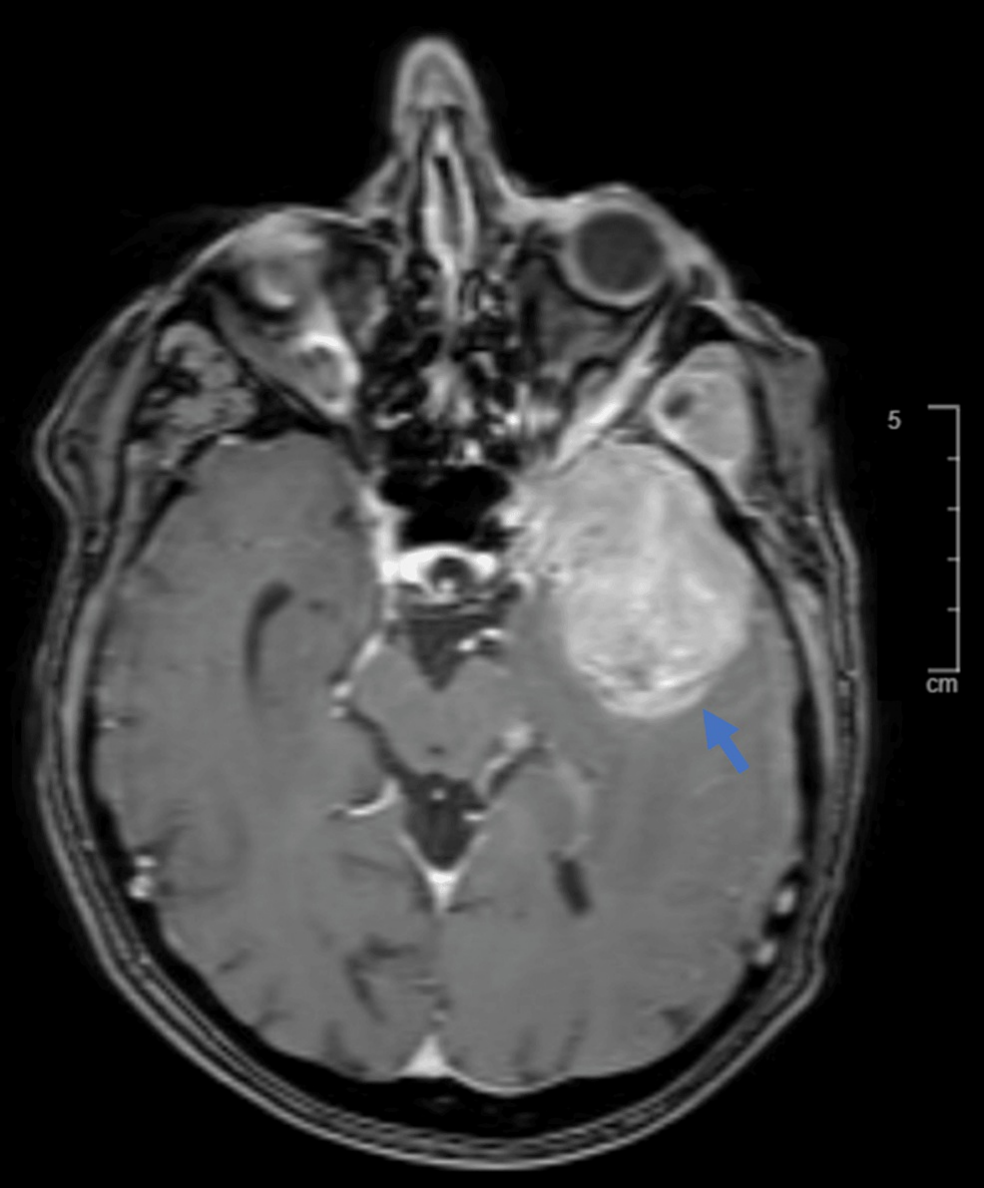
MRI scan of the brain metastasis. T1 MRI scan showed a left middle fossa brain mass (5.2 cm, blue arrow). MRI: magnetic resonance imaging.

A repeat brain MRI approximately four weeks afterward showed that the mass increased in size, measuring 5.2 cm in the greatest dimension (Figure 2). Soon after, the patient had an intracranial tumor embolization followed by resection of the left middle fossa brain mass.

**Figure 3:**
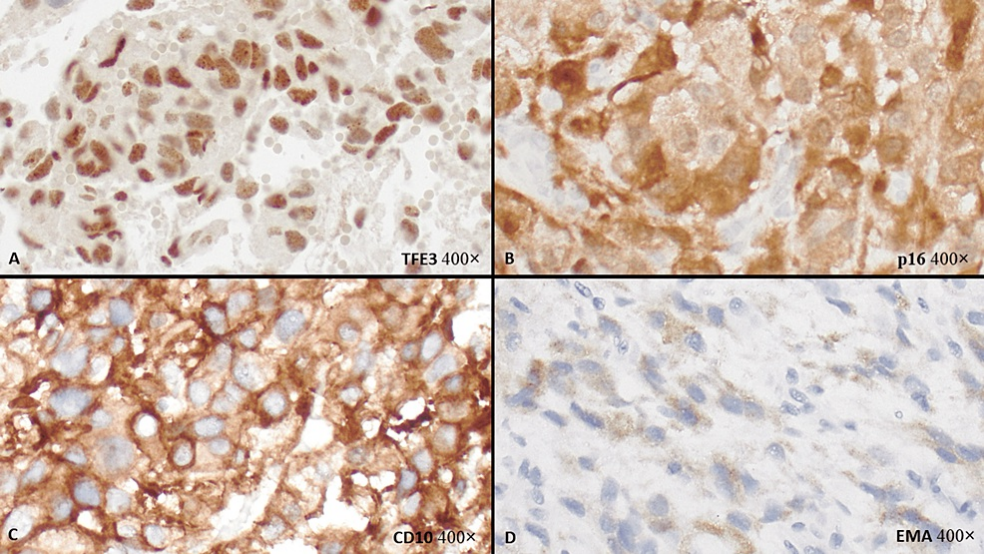
IHC stains of metastatic epithelioid pleomorphic liposarcoma in the brain. Tumor with diffuse positivity for TFE3 (A, 400x), p16 (B, 400x), and CD10 (C, 400x), and focal positivity for EMA (D, 400x). IHC: immunohistochemistry, TFE3: transcription factor enhancer 3, EMA: epithelial membrane antigen.

The resection specimen showed a high-grade malignant neoplasm with a trabecular, nested and diffuse solid proliferation of epithelioid cells separated by a collagenous matrix. The tumor had marked nuclear pleomorphism with bizarre tumor cells, atypical mitoses, focal necrosis, and multifocal lipoblastic differentiation, including numerous pleomorphic lipoblasts (Figures 1C, 1D). By IHC, the tumor cells were diffusely positive for vimentin, CD10, TFE3, and p16, focally positive for EMA (Figure 3), and negative for carbonic anhydrase IX, anti-renal cell carcinoma (RCC) antibody, PAX8, cytokeratin (CK)7, and CK20, pankeratin (AE1/AE3, osteoclast-associated receptor (OSCAR) and cytokeratin MNF116), cytokeratin CAM 5.2, S100, CD34, desmin, SMA, CD163, and MART1. FISH studies were negative for TFE3 rearrangement and MDM2 amplification. Next-generation sequencing (NGS) was negative for gene fusions involving the following genes: Anaplastic Lymphoma Kinase (ALK), Calmodulin-Binding Transcription Activator 1 (CAMTA1), Cyclin B3 (CCNB3), Capicua Transcriptional Repressor (CIC), Enhancer of Polycomb Homolog 1 (EPC1), Ewing Sarcoma Breakpoint Region 1 (EWSR1), Forkhead Box O1 (FOXO1), Fused in Sarcoma (FUS), GLI Family Zinc Finger 1 (GLI1), High Mobility Group AT-Hook 2 (HMGA2), JAZF Zinc Finger 1 (JAZF1), MYST/Esa1-Associated Factor 6 (MEAF6), Megakaryoblastic Leukemia 2 (MKL2), Nuclear Receptor Coactivator 2 (NCOA2), Platelet-Derived Growth Factor Beta (PDGFB), Pleiomorphic Adenoma Gene 1 (PLAG1), ROS Proto-Oncogene 1 (ROS1), Synovial Sarcoma Translocation, Chromosome 18 (SS18), Signal Transducer and Activator of Transcription 6 (STAT6), TATA-Box Binding Protein-Associated Factor 15 (TAF15), Transcription Factor 12 (TCF12), TFE3, TRK-Fused Gene (TFG), Ubiquitin-Specific Protease 6 (USP6), Neurotrophic Tyrosine Kinase, Receptor Type (NTRK)1, NTRK2, NTRK3, Rearranged During Transfection (RET), and Tyrosine 3-Monooxygenase/Tryptophan 5-Monooxygenase Activation Protein Epsilon (YWHAE). No Isocitrate Dehydrogenase (IDH) 1/2 and Phosphatidylinositol-4,5-Bisphosphate 3-Kinase, Catalytic Subunit Alpha (PIK3CA) mutations were identified. There was no evidence of deficient mismatch repair (MMR). Both gluteal mass and brain mass specimens were sent for outside consultations, and the final diagnoses were both epithelioid pleomorphic liposarcoma.

Two days after the surgery, a postoperative brain MRI revealed the presence of residual disease. The patient was treated with adjuvant radiation for the residual brain mass and palliative radiation therapy for the gluteal mass. Two weeks after the first radiation therapy, the patient experienced altered mental status and pain in the left eye. A brain CT scan in the emergency room showed a 2.2 cm cystic structure in the posterior left orbit producing a mass effect on the left optic nerve, which likely represented residual/recurrent neoplasm. The patient was stabilized and was discharged.

He then received additional radiation therapy without complications until he completed a total of 16 cycles of radiation therapy. A week after the last radiation therapy, the patient presented with an altered mental status and unresponsiveness. He had multi-organ failure, encephalopathy, septic shock, and multifocal pneumonia, and was deceased approximately 4.5 months post-initial presentation.

## Discussion

STS, such as LPS [8], usually exhibit a predilection for the hematogenous metastatic process, often resulting in lung metastasis [9]. In sarcoma patients with BM, approximately 55%-95% of the tumors metastasize to the lung as the prior site of disease [10,11]. Currently, there is no population-based prognostic data available about the incidence rate of BM for individual histological types of LPS. Although series and retrospective studies provide valuable information, it is essential to consider potential biases, such as referral bias, detection bias, and limited sample size, when interpreting these data [12]. PLPS has a BM rate ranging from 0 to 8.7% [6,13] in different studies. Notably, metastatic epithelioid-variant PLPS in the brain has not been reported in the literature [14,15].

In our literature search, employing the inclusion criteria of available clinicopathological data, we identified a total of five cases of brain metastases in PLPS, including our own case [16-19], as listed in Table 1. These five patients were males, aged 53 and older, with an age range of 53-76 years. Most tumors originated in the upper and lower extremities. In addition, all of them had lung metastasis as well as brain metastasis. Four patients were followed up, and they all died from this disease.

**Table 1:** PLPS cases with brain metastasis with clinicopathological data. PLPS: pleomorphic liposarcoma.

References	Age	Sex	Primary tumor	Size (cm)	Primary tumor treatment	Local recurrence	Metastasis and treatment	Prognosis
Gebhard et al. [16]	66	M	Ischiorectal fossa	15	NA	Yes, at three months	Retroperitoneum, lung, and brain metastases at 13 months (NA)	DOD at 20 months
Hornick et al. [17]	53	M	Upper arm	10	Local excision and radiation therapy	No	Lung and brain metastases at 56 months (radiation therapy)	DOD at 61 months
Yu and Sokumbi​​​​​​​ [18]	63	M	Left thigh	NA	Wide local excision and adjuvant radiotherapy	No	Lung and vertebral metastases at 12 months (chemotherapy and adjuvant radiotherapy). Scalp and brain metastasis at 36 months (palliative radiation)	DOD at 42 months
Sandhu et al. [19]	55	M	Left upper extremity	NA	Excision, radiation, and chemotherapy	NA	Lung, bone, and brain metastases, months NA (steroids and palliative total brain radiation)	Discharged, no follow-up data
This case	76	M	Right gluteus	7	Palliative radiation	No	Lung and brain metastases at diagnosis (intracranial tumor embolization, resection, adjuvant radiation, and palliative radiation)	DOD at 4.5 months
Note: NA: data not available, DOD: die of this disease								

Epithelioid-variant PLPS was initially described in 1999 by Miettinen and Enzinger [15]. This variant is characterized by epithelioid cells with abundant eosinophilic cytoplasm, honeycomb-like cell borders, and limited collagenous extracellular matrix. Ancillary tests revealed all positive for vimentin; some cases show focal positivity for keratins (AE1/AE3 and CAM 5.2), S100 [15] and A103 (a mouse monoclonal antibody against Melan-A recombinant protein) [20]. All cases were negative for EMA, SMA, desmin, CD34 [15], and alpha-inhibin [20].

Electron microscopic features of epithelioid-variant PLPS [20] include prominent organelles in the epithelioid cells of PLPS, such as mitochondria and microvesicular to coalesced lipid droplets. Notable micropinocytotic activity and inconspicuous rough endoplasmic reticulum (RER) cisternae were also observed.

The differential diagnoses of epithelioid-variant PLPS include carcinoma [21], particularly clear cell renal cell carcinoma, adrenal cortical carcinoma, melanoma [5], and other sarcoma with epithelioid morphology.

While focal epithelioid morphology appears to be associated with an increased risk of recurrence and/or metastasis and a relatively poor prognosis in PLPS [17], the occurrence of brain metastasis in the epithelioid-variant PLPS has not been reported in the literature.

In our case, the presence of pure epithelioid morphology and TFE3 expression by IHC in the initial core biopsy of the gluteal mass posed a diagnostic challenge. However, the brain resection specimen exhibited the classic morphology of epithelioid PLPS and tested negative for TFE3 gene rearrangement by FISH, as well as negativity for MDM2 amplification by FISH. These findings led to the definite final diagnosis of this case. It is important to note that the primary differential diagnosis for the brain tumor in our case is DDLPS, which has been reported in LPS cases with BM. Nevertheless, the absence of a transition from WDLPS to pleomorphic non-lipogenic morphology, coupled with the absence of MDM2 amplification as confirmed by FISH, has effectively ruled out this possibility. Our case also underscores the significance of thorough tumor sampling. Epithelioid PLPS can be missed in small biopsies; therefore, adequate sampling and a thorough search for pleomorphic lipoblasts are recommended for epithelioid neoplasms.

The finding of TFE3 overexpression by IHC but without TFE3 gene rearrangement is non-specific. It has been reported in DDLPS [22] and may be encountered in various types of neoplasms [23,24]. The tumor in our case also exhibited diffuse p16 and CD10 expression by IHC. The expressions of p16 and CD10 by IHC in PLPS are also non-specific [25,26].

Information regarding MMR deficiency in bone and soft tissue tumors is scarce. Lam et al. [27] have noted that MMR deficiency is rare in both bone and soft tissue tumors. However, it appears to be relatively frequent in soft tissue sarcomas with myogenic differentiation. In their study, all 104 cases of LPS and its subtypes, including MLPS, DDLPS, PLPS, and WDLPS, tested negative for deficient MMR. Our case of epithelioid PLPS was also negative for deficient MMR, which is consistent with the prior findings.

## Conclusions

Metastatic PLPS in the brain is a rare occurrence. The epithelioid-variant of PLPS, on the other hand, is exceedingly rare and warrants consideration in the differential diagnosis for epithelioid neoplasms. Thus, a meticulous examination of pleomorphic lipoblasts becomes crucial in such cases. The prognosis of brain metastasis of PLPS, including the epithelioid variant as in our case, is very poor. It's worth noting that TFE3 expression by IHC without TFE3 rearrangement can be observed in LPS and is non-specific.
